# Supplementary material for: A UMLS-based spell checker for natural language processing in vaccine safety
Source: BMC Med Inform Decis Mak. 2007 Feb 12;7:3. doi: 10.1186/1472-6947-7-3 (PMC1805499; doi:10.1186/1472-6947-7-3)
Supplement: Additional file 13 — RAP application directory. Sets up directory for RAP (RDF application for PHP) source code [file 1472-6947-7-3-S13.gz › rap/doc/phpdoc/__filesource/fsource_infModel__infModelInfModelF.php.html]

xml version="1.0" encoding="iso-8859-1"?


File Source for InfModelF.php


# Source for file InfModelF.php

Documentation is available at InfModelF.php

```
1. <?PHP

3. // ----------------------------------------------------------------------------------
4. // Class: InfModelF
5. // ----------------------------------------------------------------------------------


9. /**
10. * A InfModelF extends the InfModel Class, with a forward chaining algorithm.
11. * If a new statement is added, it is enferd at
12. * once and all the entailed statements are added too.
13. * When adding or removing a statement, that produced a new inference rule,
14. * all entailed statements are discarded and the whole base model is infered
15. * again.
16. * The InfModelF is safe for loops in Ontologies, that would cause infinite loops.
17. *
18. * <BR><BR>History:<UL>
19. * <LI>09-10-2004                : First version of this class.</LI>
20. * <LI>09-15-2004                : Added Index over InRule Triggers
21. *                                  to increase performance
22. *</UL>
23. * @version  V0.9.1
24. * @author Daniel Westphal <mail at d-westphal dot de>
25. *
26. * @package infModel
27. * @access    public
28. ***/

30. class InfModelF extends InfModel
31. {

33. /**
34. * Array that holds the position of the infered statements in the model
35. * @var        array
36. * @access    private
37. */
38. var $infPos;


41. /**
42. * Variable that influences the habbit when adding statements.
43. * Used by the loadModel method to increase performance.
44. * @var        boolean
45. * @access    private
46. */
47. var $inferenceEnabled;


50. /**
51. * Constructor
52. * You can supply a base_uri
53. *
54. * @param string $baseURI
55. * @access    public
56. */
57. function InfModelF($baseURI = NULL)
58. {
59. parent::InfModel($baseURI);
60. $this->infPos=array();
61. $this->inferenceEnabled=true;
62. }

64. /**
65. * Adds a new triple to the MemModel without checking if the statement
66. * is already in the MemModel.
67. * So if you want a duplicate free MemModel use the addWithoutDuplicates()
68. * function (which is slower then add())
69. * The statement is infered and all entailed statements are added.
70. *
71. * @param    object Statement    $statement
72. * @access    public
73. * @throws    PhpError
74. */
75. function add ($statement)
76. {
77. parent::add($statement);
78. if ($this->inferenceEnabled)
79. {
80. foreach ($this->entailStatement($statement) as $state)
81. {
82. //a addWithoutDublicates construct
83. if(!$this->contains($state))
84. {

86. parent::add($state);
87. //save the position of the infered statements
88. end($this->triples);
89. $this->infPos[]=key($this->triples);
90. };
91. };
92. //apply the complete inference to the model, if the added statement was able to add a rule
93. if (in_array($statement->getLabelPredicate(),$this->supportedInference))
94. $this->applyInference();
95. }
96. }


99. /**
100. * Checks if a new statement is already in the MemModel and adds
101. * the statement, if it is not in the MemModel.
102. * addWithoutDuplicates() is significantly slower then add().
103. * Retruns TRUE if the statement is added.
104. * FALSE otherwise.
105. * The statement is infered and all entailed statements are added.
106. *
107. * @param        object Statement    $statement
108. * @return    boolean
109. * @access    public
110. * @throws    PhpError
111. */
112. function addWithoutDuplicates(& $statement)
113. {
114. if(!$this->contains($statement))
115. {
116. parent::add($statement);
117. if ($this->inferenceEnabled)
118. {
119. foreach ($this->entailStatement($statement) as $statement)
120. {
121. if(!$this->contains($statement))
122. {
123. parent::add($statement);
124. //save the position of the infered statements
125. end($this->triples);
126. $this->infPos[]=key($this->triples);
127. };
128. };
129. if (in_array($statement->getLabelPredicate(),$this->supportedInference))
130. $this->applyInference();
131. }
132. return true;
133. }
134. return false;
135. }


139. /**
140. * Entails every statement and adds the entailments if not already
141. * in the model.
142. *
143. * @access    private
144. */
145. function applyInference()
146. {
147. //check every statement in the model
148. foreach ($this->triples as $statement)
149. {
150. //gat all statements, that it recursively entails
151. foreach ($this->entailStatement($statement) as $statement)
152. {
153. if (!$this->contains($statement))
154. {
155. parent::add($statement);
156. //add the InfStatement position to the index
157. end($this->triples);
158. $this->infPos[]=key($this->triples);
159. };
160. };
161. };
162. }


165. /**
166. * Entails a statement by recursively using the _entailStatementRec
167. * method.
168. *
169. * @param    object Statement    $statement
170. * @return   array of statements
171. * @access    public
172. */
173. function entailStatement (& $statement)
174. {
175. $infStatementsIndex=array();
176. return $this->_entailStatementRec($statement,$infStatementsIndex);
177. }

179. /**
180. * Recursive method, that checks the statement with the trigger of
181. * every rule. If the trigger matches and entails new statements,
182. * those statements are recursively infered too.
183. * The $infStatementsIndex array holds lready infered statements
184. * to prevent infinite loops.
185. *
186. *
187. * @param    object Statement $statement
188. * @param    array $infStatementsIndex
189. * @return   array of statements
190. * @access    private
191. */
192. function _entailStatementRec ( $statement,& $infStatementsIndex)
193. {
194. $infStatements = array();
195. $return = array();

197. //dont entail statements about the supported inference-schema
198. if (!in_array($statement->getLabelPredicate(),$this->supportedInference))
199. {
200. //check only the rules, that were returned by the index
201. foreach ($this->_findRuleTriggerInIndex($statement) as $key )
202. {
203. $infRule=$this->infRules[$key];

205. $stateString=$key.serialize($statement);
206. //If the statement wasn't infered before
207. if (!in_array($stateString,$infStatementsIndex))
208. {
209. $infStatementsIndex[]=$stateString;
210. //Check, if the Statements triggers this rule
211. if($infRule->checkTrigger($statement))
212. {
213. $infStatement=$infRule->entail($statement);
214. #if(!$this->contains($infStatement))
215. {
216. $return[]=$infStatement;
217. $return=array_merge($return,
218. $this->_entailStatementRec($infStatement,
219. $infStatementsIndex));
220. };

222. };
223. };
224. };
225. };
226. return $return;
227. }

229. /**
230. * Removes all infered statements from the model but keeps the
231. * infernece rules.
232. *
233. * @access    public
234. */
235. function removeInfered()
236. {
237. $indexTmp=$this->indexed;
238. $this->index(-1);
239. foreach ($this->infPos as $key)
240. {
241. unset($this->triples[$key]);
242. };
243. $this->infPos=array();
244. $this->index($indexTmp);
245. }


248. /**
249. * Load a model from a file containing RDF, N3 or N-Triples.
250. * This function recognizes the suffix of the filename (.n3 or .rdf) and
251. * calls a suitable parser, if no $type is given as string
252. * ("rdf" "n3" "nt");
253. * If the model is not empty, the contents of the file is added to
254. * this DbModel.
255. *
256. * While loading the model, the inference entailing is disabled, but
257. * new inference rules are added to increase performance.
258. *
259. * @param     string     $filename
260. * @param     string     $type
261. * @access    public
262. */
263. function load($filename, $type = NULL)
264. {
265. //Disable entailing to increase performance
266. $this->inferenceEnabled=false;
267. parent::load($filename, $type);
268. //Enable entailing
269. $this->inferenceEnabled=true;
270. //Entail all statements
271. $this->applyInference();
272. }

274. /**
275. * Short Dump of the InfModelF.
276. *
277. * @access    public
278. * @return    string
279. */
280. function toString() {
281. return 'InfModelF[baseURI=' . $this->getBaseURI() . ';
282. size=' . $this->size(true) . ']';
283. }

285. /**
286. * Create a MemModel containing all the triples (including inferred
287. * statements) of the current InfModelF
288. *
289. * @return object MemModel
290. * @access public
291. */
292. function & getMemModel()
293. {
294. $return= new MemModel();
295. $return->setBaseURI($this->baseURI);
296. foreach ($this->triples as $statement)
297. $return->add($statement);

299. $return->addParsedNamespaces($this->getParsedNamespaces());
300. return $return;
301. }

303. /**
304. * Create a MemModel containing only the base triples
305. * (without inferred statements) of the current InfModelF
306. *
307. * @return object MemModel
308. * @access public
309. */
310. function  getBaseMemModel()
311. {
312. $return= new MemModel();
313. $return->setBaseURI($this->baseURI);
314. foreach ($this->triples as $key => $statement)
315. if (!in_array($key,$this->infPos))
316. $return->add($statement);
317. $retun->addParsedNamespaces($this->getParsedNamespaces());
318. return $return;
319. }


322. /**
323. * Removes the triple from the MemModel.
324. * TRUE if the triple is removed.
325. * FALSE otherwise.
326. *
327. * Checks, if it touches any statements, that added inference rules
328. * to the model
329. *
330. * @param    object Statement    $statement
331. * @return   boolean
332. * @access    public
333. * @throws    PhpError
334. */
335. function remove($statement)
336. {
337. //If the statement is in the model
338. if($this->contains($statement))
339. {
340. $inferenceRulesWereTouched=false;
341. //If the statement was able to add inference rules
342. if (in_array($statement->getLabelPredicate(),$this->supportedInference))
343. {
344. $statementPositions=$this->_removeFromInference($statement);
345. $inferenceRulesWereTouched=true;
346. } else
347. //get the position of all matching statements
348. {
349. $statementPositions=array();
350. //find the positions of the statements
351. $statementPosition=-1;
352. do
353. {

355. $statementPosition =
356. $this->findFirstMatchOff($statement->getSubject(),
357. $statement->getPredicate(),
358. $statement->getObject(),
359. $statementPosition+1);

361. if ($statementPosition!=-1)
362. $statementPositions[]=$statementPosition;

364. }    while ($statementPosition != -1);
365. }

367. //remove matching statements
368. parent::remove($statement);
369. foreach ($statementPositions as $statementPosition)
370. {
371. //if the statement was infered, remove it from the index of the infered statements.
372. if (in_array($statementPosition,$this->infPos))
373. unset ($this->infPos[$statementPosition]);
374. }
375. if ($inferenceRulesWereTouched)
376. {
377. //remove the statement and re-entail the model
378. $this->removeInfered();
379. $this->applyInference();
380. }
381. return true;
382. } else
383. {
384. return false;
385. }
386. }

388. /**
389. * Adds another model to this MemModel.
390. * Duplicate statements are not removed.
391. * If you don't want duplicates, use unite().
392. * If any statement of the model to be added to this model contains a blankNode
393. * with an identifier already existing in this model, a new blankNode is generated.
394. *
395. * @param    object Model    $model
396. * @access    public
397. * @throws phpErrpr
398. *
399. */
400. function addModel(&$model)
401. {
402. //Disable entailing to increase performance
403. $this->inferenceEnabled=false;
404. parent::addModel($model);
405. //Enable entailing
406. $this->inferenceEnabled=true;
407. //Entail all statements
408. $this->applyInference();
409. }
410. };
411. ?>
```

Documentation generated on Thu, 7 Jul 2005 13:41:41 +0200 by phpDocumentor 1.3.0RC3
